# Cellular analysis and metagenomic next-generation sequencing of bronchoalveolar lavage fluid in the distinction between pulmonary non-infectious and infectious disease

**DOI:** 10.3389/fcimb.2022.1023978

**Published:** 2023-01-25

**Authors:** Yilin Pan, Xue Zhang, Yi Sun, Yingying Zhang, Wuping Bao, Dongning Yin, Pengyu Zhang, Min Zhang

**Affiliations:** ^1^ Department of Pulmonary and Critical Care Medicine, Shanghai General Hospital, Shanghai Jiao Tong University School of Medicine, Shanghai, China; ^2^ Department of Laboratory Medicine, Shanghai General Hospital, Shanghai Jiao Tong University School of Medicine, Shanghai, China; ^3^ Department of Infectious Disease, Shanghai General Hospital, Shanghai Jiao Tong University School of Medicine, Shanghai, China

**Keywords:** bronchoalveolar lavage fluid, cellular analysis, metagenomic next-generation sequencing, pulmonary non-infectious disease, pulmonary infectious disease

## Abstract

**Background:**

The aim of the current study was to investigate the clinical value of cellular analysis and metagenomic next-generation sequencing (mNGS) of bronchoalveolar lavage fluid (BALF) in differentiating pulmonary non-infectious and infectious diseases in immunocompetent patients.

**Methods:**

The present retrospective study was conducted from December 2017 to March 2020, and included immunocompetent patients with suspected pulmonary infection. High-resolution computed tomography, total cell counts and classification of BALF, conventional microbiological tests (CMTs), laboratory tests and mNGS of BALF were performed. Patients were assigned to pulmonary non-infectious disease (PNID) and pulmonary infectious disease (PID) groups based on final diagnoses. PNID-predictive values were analyzed *via* areas under receiver operating characteristic curves (AUCs). Optimal cutoffs were determined by maximizing the sum of sensitivity and specificity.

**Results:**

A total of 102 patients suspected of pulmonary infection were enrolled in the study, 23 (22.5%) with PNID and 79 (77.5%) with PID. The diagnostic efficiency of BALF mNGS for differentiating PID from PNID was better than that of CMTs. Neutrophil percentage (N%) and the ratio of neutrophils to lymphocytes (N/L) in BALF were significantly lower in the PNID group than in the PID group. The AUCs for distinguishing PNID and PID were 0.739 (95% confidence interval [CI] 0.636–0.825) for BALF N%, 0.727 (95% CI 0.624–0.815) for BALF N/L, and 0.799 (95% CI 0.702–0.876) for BALF mNGS, with respective cutoff values of 6.7%, 0.255, and negative. Joint models of BALF mNGS combined with BALF N/L or BALF N% increased the respective AUCs to 0.872 (95% CI 0.786–0.933) and 0.871 (95% CI 0.784–0.932), which were significantly higher than those for BALF mNGS, BALF N%, and BALF N/L alone.

**Conclusions:**

BALF N% ≤ 6.7% or BALF N/L ≤ 0.255 combined with a negative BALF mNGS result can effectively distinguish PNID from PID in immunocompetent patients with suspected pulmonary infection. BALF mNGS outperforms CMTs for identifying pathogens in immunocompetent patients, and the combination of mNGS and CMTs may be a better diagnostic strategy.

## 1 Introduction

Pulmonary infection is one of the most common reasons for hospitalization (Rana et al., 2021). However, a considerable number of patients who are initially diagnosed with pulmonary infectious disease (PID) do not exhibit any signs of recovery after treatment with various antibiotics, and their condition ultimately proves to be a pulmonary non-infectious disease (PNID) (Flament et al., 2016; Cottin and Brown, 2019; Fujita et al., 2020). Patients with PID and PNID usually have similar symptoms and physical signs (Allen and Wert, 2018; Raghu and Meyer, 2021), making it difficult to distinguish between the two based on inquiry and physical examination alone, without auxiliary examinations.

The treatment principles of PID and PNID are quite different. Misdiagnosis can lead to a prolonged recovery time, or worse, result in irreparable losses (Cosgrove et al., 2018). Prescribing antibiotics to patients with PNID leads to abuse-grade resistance, and prolongs the patient’s condition. Rapid and accurate identification of PID and PNID is therefore crucial.

Clinical parameters such as routine blood test results, C-reactive protein (CRP), procalcitonin (PCT), and erythrocyte sedimentation rate (ESR) are often used to assist determination of the severity of infection (Wacker et al., 2013; Prina et al., 2015; Sproston and Ashworth, 2018; Lapic et al., 2020). They can also be used to predict pulmonary infection, and are relatively easy to obtain, particularly in primary settings. However, their predictive role with respect to infectious diseases remains controversial (Ito and Ishida, 2020; Niu et al., 2021).

Bronchoscopy facilitates the examination of airways, and the acquisition of bronchoalveolar lavage fluid (BALF). Bronchoscopy is a recommended method for the diagnosis of PID (Metlay et al., 2019). Early bronchoscopy in immunocompromised patients with pulmonary infiltrates can reportedly improve outcomes (Gonski et al., 2020; Bourne et al., 2021; Saksirisampant et al., 2022). Cellular analysis of BALF is simple and safe, and can reflect some underlying features of disease. Kono et al. (Kono et al., 2021) reported that the ratio of lymphocytes to neutrophils (N/L) in BALF can predict the prognosis of acute exacerbation of interstitial lung disease. BALF can also be used for smears, cultures, and metagenomic next-generation sequencing (mNGS) to identify PID pathogens. Compared with conventional microbial detection methods such as smears and cultures, mNGS is evidently superior with regard to diagnostic efficiency (Wilson et al., 2014; Zhou et al., 2016; Takeuchi et al., 2019; Huang et al., 2020). While most studies emphasize the sensitivity of mNGS, however, its excellent specificity is rarely mentioned.

The aim of the current study was to determine whether cellular analysis and mNGS of BALF, and other infection indexes alone or in different combinations, could differentiate between PID and PNID in immunocompetent patients; and therefore enhance the accuracy of clinical diagnoses.

## 2 Methods

### 2.1 Participants and study design

Patients who presented with suspected pulmonary infections at Shanghai General Hospital in Shanghai, China, from December 2017 to March 2020 were retrospectively reviewed. The Ethics Committee of Shanghai General Hospital, Shanghai Jiao Tong University School of Medicine approved the protocol (2021KY066). Due to the retrospective nature of the study, the requirement for written informed consent was waived.

The inclusion criteria were (1) suspected pulmonary infection based on at least one compatible symptom such as new-onset fever, cough, or dyspnea, and new-onset high-resolution computed tomography (HRCT) findings on chest images; (2) the performance of bronchoscopy and bronchoalveolar lavage (BAL), and consent to perform BALF mNGS; (3) BALF and other relevant samples available for standard procedures and BALF mNGS within 48 h after admission; and (4) complete medical data recorded. The exclusion criteria were (1) the presence of an immunosuppressive condition, which was defined as suffering from any of the following: 1) primary immune deficiency diseases; 2) active malignancy excluding patients with early-stage cancers (eg, stage 1 lung cancer); 3) receiving cancer chemotherapy; 4) HIV infection with a CD4 T-lymphocyte count < 200 cells/μL or percentage < 14%; 5) solid organ transplantation; 6) hematopoietic stem cell transplantation; 7) receiving corticosteroid therapy with a dose ≥ 20 mg prednisone or equivalent daily for ≥ 14 d or a cumulative dose > 600 mg of prednisone; 8) receiving biological immune modulators; 9) receiving disease-modifying antirheumatic drugs or other immunosuppressive drugs (eg, cyclosporin, cyclophosphamide, hydroxychloroquine, methotrexate) (Ramirez et al., 2020); (2) BALF samples or detection process failed to pass quality control for mNGS; (3) repeated enrollment of the same patient; and (4) incomplete medical history.

A total of 102 patients were included in the analysis, and categorized into two groups based on final diagnoses: PNID and PID. Samples were subjected to conventional microbiological tests (CMTs), and mNGS of BALF was conducted in a pairwise manner. The CMTs used in this study are detailed in **Table S1**, and were conducted in accordance with previous studies (Parize et al., 2017; Peng et al., 2021). They including culture, serological diagnosis, antigen detection, PCR, and direct microscopic examination of specimens. The results of CMTs were interpreted in accordance with standard procedures (Patterson et al., 2016; Azoulay et al., 2020). The parameters assessed as infection indices included routine blood tests, high-sensitivity CRP (hs-CRP), PCT, interleukin (IL) 6, endotoxin, and ESR.

### 2.2 Fiberoptic bronchoscopy

Fiberoptic bronchoscopy was performed within 2 days after the identification of pulmonary infiltrates *via* HRCT. Most patients underwent fiberoptic bronchoscopy in the bronchoscopy unit. Some were performed at the bedside in the intensive care unit. BALF sampling was performed by experienced physicians in accordance with the American Thoracic Society (ATS) operating guidelines. All BALF samples were obtained from the area of lung infiltration. If there were multiple areas of infiltration, the sample was obtained where the infiltration was most severe. All BALF samples were > 20 mL in volume.

### 2.3 BALF cell count and cell classification

BALF samples were mixed 10–20 times, then approximately 5–10 μL of the sample was loaded into a Neubauer counting plate. After standing for 1 min, the number of cells was counted. The remainder of the sample was centrifuged at 400 *g*/min for 10 mins. After discarding the supernatant, the precipitate was pipetted onto a glass slide, spread evenly, then air-dried naturally. The slides were then Wright Giemsa stained. A site with a uniform distribution of cells was then selected by an experienced cytomorphologist under light microscopy, and at least 200 cells were counted and classified as neutrophils, lymphocytes, eosinophils, basophils, macrophages, ciliated columnar cells, or epithelium. Classification results were recorded as percentages.

### 2.4 mNGS

#### 2.4.1 Sample processing and nucleic acid extraction

BALF samples were collected in accordance with standard procedures. DNA was extracted from the samples using the TIANamp Micro DNA Kit (DP316; Tiangen Biotech, Beijing, China) in accordance with the manufacturer’s protocol.

#### 2.4.2 Construction of DNA libraries

Single-stranded DNA (ssDNA) libraries were constructed after DNA fragmentation, end repair, adapter ligation, denaturation into single strands, and circularization. DNA nanoballs were generated from ssDNA by rolling circle amplification, loaded into the flow cell, and sequenced on a BGISEQ-200 platform (BGI, Beijing, China) and a NextSeq 550 platform (Illumina, California, USA) (Jeon et al., 2014; Miller et al., 2019).

#### 2.4.3 Sequencing and bioinformatic analysis

High-quality sequencing data were generated by removing low-quality and short-length (< 35-bp) reads, followed by a computational subtraction of human sequences mapped to the human reference genome (hg19) *via* Burrows–Wheeler alignment (Li and Durbin, 2009). After removing low-complexity reads, the remaining data were classified *via* simultaneous alignment with four NCBI microbial genome databases (ftp://ftp.ncbi.nlm.nih.gov/genomes/). These databases included whole genome sequences of 4061 viral taxa, 2473 bacterial genomes or scaffolds, and genomic sequences for 199 fungi related to human infection and 135 parasites associated with human diseases.

#### 2.4.4 Criteria for a positive mNGS result

Given the lack of a standard method for interpreting mNGS results and the variety of reporting parameters amongst different sequencing platforms, we used the following criteria in this study, which was derived and revised from prior literature (Qian et al., 2020; Peng et al., 2021), to define clinically significant microbes.

The sequencing results of each sample were categorized into 2 tables, each presenting bacteria, fungi and virus, respectively. The specifically mapped read number (SMRN) of each microbial taxonomy was normalized to SMRN per 20 million (M) of total sequencing reads (SDSMRN, standardized SMRN).

SDSMRN = SMNTotal reads × 20 million

A bacterial/fungal species was considered positively detected if: 1) it belonged to the top 10 genera with the highest SDSMRN; 2) it ranked first within its genus; 3) it had a SDSMRN>1; and 4) it was a commonly reported pulmonary infectious pathogen. A virus was considered positively detected if: 1) it was among the top 3 viruses with highest SDSMRN; and 2) it had a SDSMRN > 5.

There were several exceptions for certain pathogens. Because the possibility of Mycobacterium spp., Nocardia spp., etc. contamination and yield rate were low, they were considered positively detected when the SMRN at the species level was more than 3. Given the balance of environmental contamination and the difficulty of DNA extraction, molds, including Aspergillus spp., Rhizopus spp., and Mucor spp., with literature-proven pulmonary pathogenicity, were considered positively when the SMRN at the species level was more than 10.

### 2.5 Diagnosis of pulmonary infections

The final diagnosis was made by two intensivists with expertise in the management of infection after independently reviewing the medical records including clinical manifestations, laboratory tests, chest HRCT, microbiological tests (including CMTs and BALF mNGS), and treatment responses. Any disagreement between the two intensivists was resolved by in-depth discussion, and another senior intensivist was consulted if a consensus could not be reached.

### 2.6 Statistical analysis

Data analyses were performed using SPSS version 24.0 (SPSS Inc., Chicago, Illinois, USA) and R version 4.1.1 (Innovative Solutions, St. Louis, MO, USA). Baseline data are presented descriptively. Normality of data distributions was assessed with the Kolmogorov–Smirnov test. Normally distributed data are presented as means ± standard deviation. Non-normally distributed data are presented as medians and interquartile ranges (IQRs). Comparisons between patients with and without infection or organizing pneumonia were performed *via* the Mann-Whitney U test. With the final diagnosis as the gold standard, the conventional method and BALF mNGS were analyzed and compared. Comparative analysis was conducted *via* the Pearson χ^2^ test, Fisher’s exact test, or the McNemar test for discrete variables where appropriate. Receiver operating characteristic (ROC) analysis was performed to determine the optimal cutoff for BALF cell patterns for infection or organizing pneumonia diagnosis. *p*<0.05 was considered to indicate statistically significant differences.

## 3 Results

### 3.1 Demographic and clinical characteristics

A total of 102 patients with suspected pulmonary infection were included in the final analysis. There were 23 patients in the PNID group (22.5%), and 79 patients in the PID group (77.5%) (**Table 1**). No demographic factors differed significantly in the two groups. Most patients received empiric antibiotic therapy before admission. Conventional infection indicators such as routine blood test results, hs-CRP, PCT, IL-6, endotoxin, and ESR did not differ significantly in the two groups. With respect to HRCT findings there were no significant differences in bilateral lesions, consolidation, ground-glass opacities, solid nodules, tree-in-bud infiltrates, atelectasis, or cavities. Fourteen patients in the PID group had bronchiectasis, and no patients in the PNID group had bronchiectasis (*p*>0.05).

**Table 1:** Demographic data and cytometry and infectious laboratory parameters in noninfectious pulmonary disease and infectious pulmonary disease.

	Non-infectious pulmonary disease (*n* = 23)	Infectious pulmonary disease (*n* = 79)	*p* value
**Age (years)¶**	57.00 (13.00)	59.00 (16.00)	0.416
**Male, *n* (%)**	9 (39.13%)	41 (51.90%)	0.166
**Empirical use of antibiotics, *n* (%)**	21 (91.30%)	68 (86.07%)	0.233
Laboratory findings			
**Leukocytes (10^9^/L)¶**	7.02 (4.32)	8.21 (3.29)	0.086
**Neutrophils (10^9^/L)¶**	4.82 (3.36)	3.52 (2.88)	0.097
**Neutrophils (%)§**	66.42 ± 9.31	64.39 ± 12.13	0.171
**Lymphocytes (10^9^/L)¶**	1.64 (0.85)	1.44 (0.73)	0.151
**Lymphocytes (%)§**	24.56 ± 8.28	25.53 ± 10.79	0.211
**Neutrophils/lymphocytes¶**	2.49 (1.81)	2.73 (2.18)	0.770
**Eosinophils (10^9^/L)¶**	0.15 (0.17)	0.13 (0.13)	0.706
**Eosinophils (%)¶**	1.90 (2.30)	2.20 (2.20)	0.715
**hs-CRP (mg/L)¶**	3.5 (18.50)	7 (25.58)	0.521
**PCT (ng/mL)¶**	0.037 (0.039)	0.037 (0.033)	0.983
**IL-6 (pg/mL)¶**	3.98 (38.79)	6.60 (12.69)	0.812
**Endotoxin (EU/mL)¶**	0.027 (0.041)	0.032 (0.055)	0.368
**ESR (mm/h)¶**	22.5 (72.5)	23.5 (40.0)	0.996
HRCT findings			
**Bilateral pulmonary lesions, *n* (%)**	16.00 (69.57%)	44 (55.70%)	0.234
**Consolidation, *n* (%)**	13 (56.52%)	48 (60.76%)	0.715
**Ground-glass opacities, *n* (%)**	7 (30.43%)	13 (16.46%)	0.235
**Solid nodules, *n* (%)**	6 (26.09%)	15 (18.99%)	0.654
**Bronchiectasis, *n* (%)**	0 (0.00%)	14 (17.72%)	0.067
**Tree-in-bud infiltrates, *n* (%)**	2 (8.70%)	7 (8.86%)	1.000
**Atelectasis, *n* (%)**	1 (4.35%)	4 (5.06%)	1.000
**Cavities, *n* (%)**	3 (13.04%)	14 (17.72%)	0.832

hs-CRP, high-sensitivity C-reactive protein; PCT, procalcitonin; IL-6, interleukin 6; ESR, erythrocyte sedimentation rate; HRCT, high-resolution computed tomography.

§ Mean ± standard deviation values.

¶ Median (interquartile range).

Statistical significance is shown by bold font.

### 3.2 Distributions of pulmonary infectious pathogens and non-infectious diseases

Among the 79 patients with PID, bacteria were the most common cause of infection (**Table 2**). *Mycobacterium tuberculosis* (13.9%) was the most commonly detected pathogen, followed by nontuberculosis mycobacteria (10.1%). Fungal infections occurred in 13.9% of patients, and the most common were *Aspergillus* and *Cryptococcus neoformans*. One patient was infected with cytomegalovirus, and three were infected with *Mycoplasma pneumoniae*. Limited by the sensitivity of current diagnostic techniques and disease progression, there were 26 patients whose specific pathogen was uncertain. The etiologies of PNID are shown in **Table 3**. The most common was organizing pneumonia (56.5%), followed by lung cancer (17.4%) and eosinophilic pneumonia (13.1%). These three are often mistaken for pneumonia (Rothberg, 2022).

**Table 2:** Distribution of pathogens identified in patients with infectious pulmonary disease.

Pathogen	*n* (%)^*^
**Bacteria**	43 (54.4%)
*Mycobacterium tuberculosis*	11 (13.9%)
*Nontuberculosis mycobacteria*	8 (10.1%)
*Hemophilus parainfluenzae*	5 (6.3%)
*Pseudomonas aeruginosa*	4 (5.1%)
*Klebsiella pneumoniae*	4 (5.1%)
*Enterococcus faecium*	2 (2.5%)
*Moraxella catarrhalis*	2 (2.5%)
*Nocardia*	2 (2.5%)
Other bacteria^#^	5 (6.3%)
**Fungi**	10 (12.7%)
*Aspergillus* spp.	4 (5.1%)
*Cryptococcus neoformans*	4 (5.1%)
*Candida* spp.	2 (2.5%)
**Viruses**	1 (1.3%)
Cytomegalovirus	1 (1.3%)
**Atypical pathogens**	3 (3.8%)
*Mycoplasma pneumonia*	3 (3.8%)

^*^Number of patients, with the percentage in parentheses.

^#^Including Staphylococcus aureus (n = 1); Acinetobacter lwoffii (n = 1); Tropheryma whipplei (n = 1); Streptococcus constellatus (n = 1); Enterobacter cloacae (n = 1).

**Table 3:** Distribution of non-infectious pulmonary diseases.

Disease	*n* (%)^*^
Organizing pneumonia	13 (56.5%)
Eosinophilic pneumonia	3 (13.1%)
Bronchiolitis	2 (8.7%)
Lung cancer	4 (17.4%)
Still’s disease	1 (4.3%)

^*^Number of patients, with the percentage in parentheses.

### 3.3 Comparison of BALF mNGS and CMTs

#### 3.3.1 Comparison of diagnostic performance for differentiating PID from PNID

**Figure 1:**
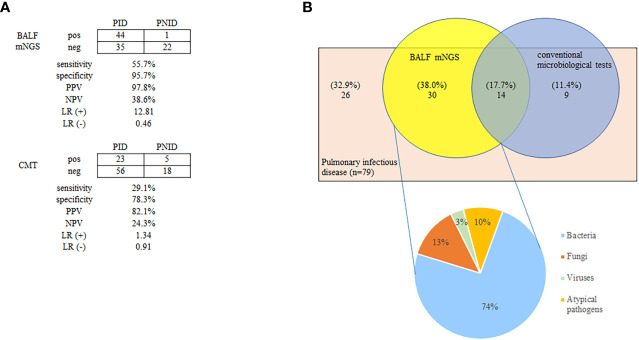
Positivity rates of BALF mNGS and CMTs for pulmonary infectious and non-infectious diseases **(A)**, and diagnosis assisted by BALF mNGS for PID without identifiable etiology by CMTs **(B)**. The positive predictive value of BALF mNGS for the diagnosis of infectious disease was 97.8%, and the negative predictive value was 38.6%. BALF mNGS increased the sensitivity rate by approximately 27% in comparison with CMTs (55.7% vs. 29.1%; *p* < 0.01) **(A)**. Thirty patients (38.0%) were BALF mNGS-positive despite comprehensive CMTs being negative. Bacteria constituted the highest proportion of pathogens (74%) **(B)**. BALF, bronchoalveolar lavage fluid; CMT, conventional microbiological test; LR(+), positive likelihood ratio; LR(-), negative likelihood ratio; mNGS, metagenomic next-generation sequencing; NPV, negative predictive value; PPV, positive predictive value; PID, pulmonary infectious disease; PNID, pulmonary non-infectious disease; ROC, receiver operating characteristic.

The positivity rates of BALF mNGS and CMTs for pulmonary infectious and noninfectious disease are shown in **Figure 1A**. The positive predictive value for diagnosing infectious disease *via* BALF mNGS was 97.8%, and the negative predictive value was 38.6%. The positive likelihood ratio was 12.81, and the negative likelihood ratio was 0.46. BALF mNGS increased the sensitivity rate by approximately 27% in comparison with CMTs (55.7% vs. 29.1%, *p*<0.01), whereas there was no significant difference in specificity (95.7% vs. 78.3%, *p*>0.1) (**Figure 1A**). Additional details are provided in **Table S2**.

#### 3.3.2 Diagnosis assisted by BALF mNGS for PID without identifiable etiology by CMTs

Among the 79 patients diagnosed with pulmonary infectious diseases, 44 were confirmed by BALF mNGS and 23 were confirmed by CMTs. Notably 30 (38.0%) were BALF mNGS-positive despite comprehensive CMTs being negative. Bacteria accounted for the highest proportion of confirmed pathogens (74%) (**Figure 1B**). Antibiotics use was adjusted in these 30 patients based on the results of BALF mNGS, and most patients improved.

### 3.4 Findings of BALF cellular analysis

BAL was performed safely in all patients, irrespective of whether the patient was intubated. N% and N/L in BALF were significantly lower in the PNID group (both *p*<0.01) (**Table 4**).

**Table 4:** Characteristics of BALF cellular analysis in non-infectious pulmonary disease and infectious pulmonary disease.

	Non-infectious pulmonary disease (*n* = 20)	Infectious pulmonary disease (*n* = 71)	*p* value
**BALF karyocytes (10^6^/L)¶**	280 (330)	280 (621)	0.562
**BALF lymphocytes (%)¶**	11.65 (47.40)	8.00 (21.00)	0.087
**BALF neutrophils (%)¶**	4.00 (10.75)	37.50 (72.00)	0.001
**BALF neutrophils/lymphocytes¶**	0.25 (2.17)	3.57 (16.92)	0.002

Statistical significance is indicated by bold font.

BALF, bronchoalveolar lavage fluid.

¶ Median (interquartile range).

### 3.5 Diagnostic accuracy of single measurements used for PNID prediction

**Figure 2:**
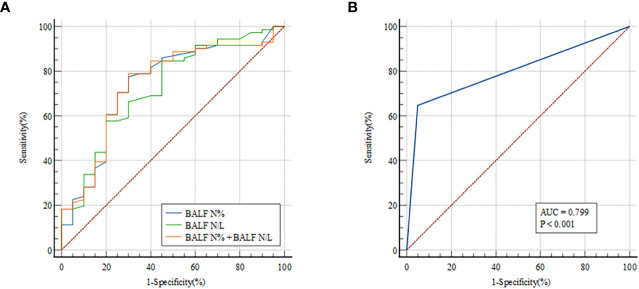
ROC curves for predicting PNID with single measurements. AUCs were 0.739 (95% CI 0.636–0.825) for BALF N% **(A)**, 0.727 (95% CI 0.624–0.815) for BALF N/L **(A)**, 0.742 (95% CI 0.640–0.828) for BALF N% combined with BALF N/L **(A)**, and 0.799 (95% CI 0.702–0.876) for BALF mNGS **(B)**. AUC, area under the curve; BALF, bronchoalveolar lavage fluid; mNGS, metagenomic next-generation sequencing; N, neutrophils; N/L, ratio of neutrophils to lymphocytes; PNID, pulmonary non-infectious disease; ROC, receiver operating characteristic.

There were significant differences in N% and N/L in BALF between patients with and without infectious disease, indicating that these measurements may predict PNID. Therefore, ROC curves were constructed to evaluate the prognostic value of these measurements for PNID, and AUCs were calculated. In ROC analysis of BALF N% the AUC was 0.739 (95% CI: 0.636–0.825), and the AUC of BALF N/L was 0.727 (95% CI: 0.624–0.815) (**Figure 2A**). The AUC of BALF N% and BALF N/L combined was 0.742 (95% CI: 0.640–0.828), which was not significantly higher than that of BALF N% alone. The AUC of BALF mNGS for PNID prediction was 0.799 (95% CI: 0.702–0.876) (**Figure 2B**).

### 3.6 Diagnostic accuracy of BALF cellular analysis combined with mNGS for PNID prediction

**Figure 3:**
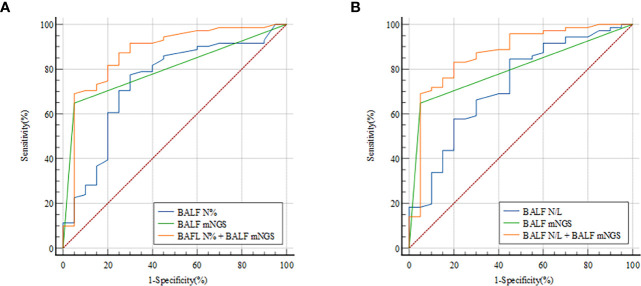
ROC curves for predicting PNID with BALF cellular analysis combined with mNGS. AUCs were 0.872 (95% CI 0.786–0.933) for BALF N% combined with BALF mNGS **(A)** and 0.871 (95% CI 0.784–0.932) for BALF N/L combined with BALF mNGS **(B)**. AUC, area under the curve; BALF, bronchoalveolar lavage fluid; mNGS, metagenomic next-generation sequencing; N, neutrophils; N/L, ratio of neutrophils to lymphocytes; PNID, pulmonary non-infectious disease; ROC, receiver operating characteristic.

To determine whether combining measurements would improve PNID prediction binary logical regression of BALF N% and BALF mNGS was performed, and it yielded an AUC of 0.872 (95% CI: 0.786–0.933) (**Figure 3A**), which was significantly higher than those of BALF N% alone and BALF mNGS alone (both *p*<0.05). In ROC analysis of BALF N/L combined with BALF mNGS the AUC was 0.871 (95% CI: 0.784–0.932) (**Figure 3B**), which was significantly higher than that of BALF mNGS alone and BALF N/L alone (both *p*<0.05).

### 3.7 Optimal cutoff values for PNID

**Figure 4:**
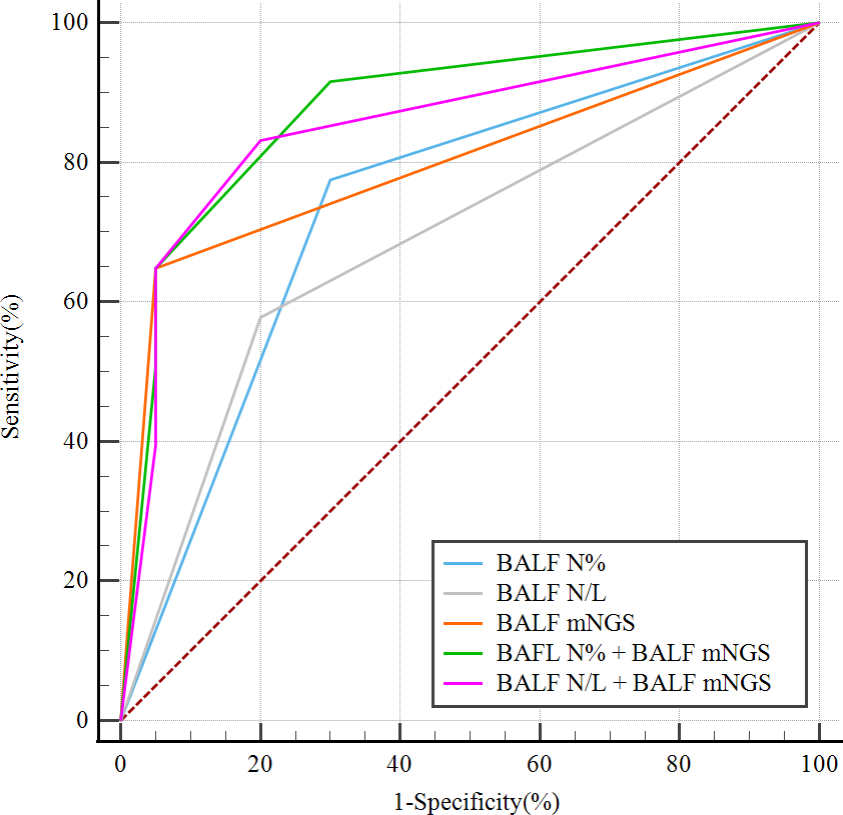
ROC curves of dichotomous state variables of BALF mNGS combined with BALF N% (model 1) or BALF N/L (model 2) for predicting PNID, compared with single variables alone. Dichotomous state variables were separated in accordance with cutoff values of BALF mNGS, BALF N%, and BALF N/L. AUC_model 1_ = 0.879 (95% CI 0.793–0.938); AUC_model 2_ = 0.853 (95% CI 0.763–0.919); AUC_BALF N%_ = 0.737 (95% CI 0.635–0.824); AUC_BALF N/L_ = 0.689 (95% CI 0.583–0.782); AUC_BALF mNGS_ = 0.799 (95% CI 0.702–0.876). AUC, area under the curve; BALF, bronchoalveolar lavage fluid; mNGS, metagenomic next-generation sequencing; N, neutrophils; N/L, ratio of neutrophils to lymphocytes; PNID, pulmonary non-infectious disease; ROC, receiver operating characteristic.

Optimal BALF N%, BALF N/L, and BALF mNGS cutoff values for predicting PNID were calculated as the values that gave the highest sum of sensitivity and specificity in the immunocompetent patients. The cutoff values for the measures with the highest AUCs were 7% for BALF N%, 0.25 for BALF N/L, and negative for BALF mNGS results. Using the cutoff values, the continuous test variables were converted to dichotomous state variables by defining BALF N%≤6.7% as “1”, BALF N%>6.7% as “0”, BALF N/L ≤ 0.255 as “1”, BALF N/L>0.255 as “0”, a negative BALF mNGS result as “1”, and a positive BALF mNGS result as “0”. Logistic regression was then performed to generate a predictive equation based on the combination of measurements, followed by multiple logistic regression analyses of BALF N%, BALF N/L, BALF mNGS, BALF N% combined with BALF mNGS (model 1), and BALF N/L combined with BALF mNGS (model 2). The AUC of model 1 was 0.879 (95% CI: 0.793–0.938), the AUC of the model 2 was 0.853 (95% CI: 0.763–0.919), and the AUCs of BALF N%, BALF N/L, and BALF mNGS were 0.737 (95% CI: 0.635–0.824), 0.689 (95% CI: 0.583–0.782), and 0.799 (95% CI: 0.702–0.876), respectively (**Figure 4**).

## 4 Discussion

To our knowledge, our research pioneered the use of the combination of BALF cellular analysis and BALF mNGS to distinguish between PNID and PID. The joint models of BALF N%≤6.7% and BALF N/L ≤ 0.255 combined with a negative BALF mNGS result can predict PNID, and may provide a rapid and precise method for physicians to differentiate between non-infectious and infectious diseases. Compared with CMTs, BALF mNGS had higher diagnostic efficiency and effectively filled the gap when CMT evidence was insufficient.

Pulmonary infection is one of the most common types of infection, and a common cause of hospitalization and death in older adults (Global Burden of Disease Study, 2015). Patients with suspected pulmonary infection who require hospitalization tend to have more complicated conditions (Postma et al., 2017). In the current study more than half the patients exhibited a multilobar distribution of pleomorphic lesions on HRCT, and the vast majority had administered antibiotics before admission. Among the patients diagnosed with pulmonary infection, more than 20% had mycobacterial infections. All these factors indicated that it was not simply a case of community-acquired pneumonia. Given the complexity of hospitalized patients, the diagnoses of PNID and PID were based on clinical manifestations, laboratory tests, chest HRCT, CMTs, BALF mNGS, and treatment responses.

Routine blood tests are the most commonly used diagnostic method, and can reflect the severity of infection to an extent. Physicians in primary hospitals usually diagnose infections based on the results of routine blood tests. In the present study there were no significant differences in routine blood test indexes between the PNID group and the PID group, which is consistent with previous studies (Zhu et al., 2015; Shaddock, 2016). CRP, PCT, and ESR are clinically validated indicators that can be used to assist the diagnosis of infection (Wang et al., 2019; Yin and Mo, 2022). However, many non-infectious conditions such as allergies and autoimmune diseases can dramatically influence CRP and ESR values (Giacomelli et al., 2018; Davis and van der Hilst, 2018; Aringer, 2020), whereas PCT does not change significantly in some Gram-positive bacterial and fungal infections or local infections (Ito and Ishida, 2020; Xu et al., 2021). In the current study these three indicators were poor for distinguishing between PNID and PID. This may be related to the empirical use of antibiotics before admission. The same results were also evident with respect to emerging indicators for infection assessment such as IL-6 and endotoxin. The principles of treatment for non-infectious diseases and infectious diseases are quite different, and patients with suspected pulmonary infection requiring hospitalization tend to have complex medical conditions. Inappropriate treatment can lead to prolonged disease, antibiotic abuse, and even death. It is therefore important to identify an effective method to distinguish PNID from PID.

BAL is a simple and safe procedure. BALF is obtained from deep bronchi which are relatively sterile, and it contains significantly less background microorganisms than sputum, so it can better reflect the local microenvironment of the lungs, and is more suitable for culture and mNGS. The proportion of lymphocytes in BALF can reportedly be used as a prognostic predictor of acute exacerbation of interstitial lung disease (Takei et al., 2017; Kono et al., 2021). The proportion of eosinophils in BALF can constitute evidence for the diagnosis of eosinophil-associated pneumonia and parasitic infection (Ravin and Loy, 2016; Allen and Wert, 2018). Notably however, few studies have investigated whether cellular analysis of BALF can be used to distinguish between PID and PNID. In the current study BALF N% and BALF N/L could effectively differentiate between PNID and PID, and a possible explanation is that neutrophils in BALF are closer to the pulmonary lesions and more capable of responding to local infection and inflammation of lung. Bronchoscopy is now available in most hospitals, and as a safe and convenient examination BAL is receiving increasing attention from respiratory specialists. We recommend routine BAL to help distinguish between PNID and PID, especially in patients with severe illness.

mNGS is an unbiased detection method that can theoretically detect all pathogens in clinical samples (Chiu and Miller, 2019). In this study, the sensitivity of BALF mNGS for detecting pathogens was 55.7% and the sensitivity of CTMs was 29.1%. Most of the patients with typical symptoms were effectively treated in the outpatient department, while the patients hospitalized in tertiary hospitals for pulmonary infection tended to have more complicated conditions and most of them had been empirically treated with broad-antibiotics. 87.3% of the patients included in our study had received empirical antibiotics before admission. In the context of prior antibiotic application, the sensitivity of CMTs is significantly lower than that of mNGS. Our results are consistent with previous study (Miao et al., 2018). To date most research has focused on the ability of mNGS to detect pathogens, and has emphasized its sensitivity. Conversely mNGS is limited by interference from background microorganisms and contamination, so results may be false-positives, leading to overdiagnosis (Simner et al., 2018). However, this feature means that negative mNGS results are more credible. In the present study negative BALF mNGS results were used to exclude pulmonary infection in order to screen for PNID. BALF mNGS could effectively predict PNID, and its diagnostic efficiency was improved when it was combined with cellular BALF analysis. Therefore, we recommend that BALF mNGS should be conducted when economic considerations permit or the patient is severely ill, to help physicians make correct diagnoses in a short time and formulate appropriate treatment plans.

The current study had some limitations. It was a single-centered study with a relatively small sample size, therefore multicenter, large-scale, prospective studies are needed in the future to validate the results. Immunocompetent patients were recruited rather than immunosuppressed patients, which may be associated with significantly different pathogen profiles, as well as diverse diagnostic performance of mNGS and cellular analysis of BALF. Lastly, the lack of RNA sequencing and insufficient PCR methods hindered evaluation of the diagnostic value of CMTs and mNGS with respect to virus infection.

In conclusion, the current study indicates that BALF N% ≤ 6.7% or BALF N/L ≤ 0.255 combined with a negative BALF mNGS result can effectively distinguish PNID from PID in immunocompetent patients with suspected pulmonary infection. It may help physicians to determine the optimal treatment, and avoid the abuse of antibiotics. With regard to primary hospitals without access to complex equipment, BAL is a safe, convenient, and economical method that can improve the accuracy of PNID and PID diagnoses. BALF mNGS outperforms CMTs with respect to identifying pathogens in immunocompetent patients, and the combination of mNGS and CMTs may be a better diagnostic strategy.

## Data availability statement

The data presented in the study are deposited in CNGB Sequence Archive (CNSA) of China National GeneBank DataBase (CNGBdb), accession number CNP0003771.

## Ethics statement

The studies involving human participants were reviewed and approved by The ethics committee of Shanghai General Hospital, Shanghai Jiao Tong University School of Medicine. The ethics committee waived the requirement of written informed consent for participation.

## Author contributions

MZ, PZ, and YP conceived of and designed the entire study. YZ, YP, WB, and DY contributed to acquisition of data. YP, XZ, and YS performed statistical analyses. MZ, PZ, and YP were involved in the interpretation of data. YP, XZ, and YS wrote the manuscript, supervised by MZ. All authors contributed to the article and approved the submitted version. All authors agree to be accountable for all aspects of the work in ensuring that questions related to the accuracy or integrity of any part of the work are appropriately investigated and resolved.

## Supplementary material

Supplementary Table 1 Conventional microbiological methods used in the study.

Supplementary Table 2 Sample details of enrolled cases.
