# Supplementary material for: Cellular analysis and metagenomic next-generation sequencing of bronchoalveolar lavage fluid in the distinction between pulmonary non-infectious and infectious disease
Source: Front Cell Infect Microbiol. 2023 Jan 25;12:1023978. doi: 10.3389/fcimb.2022.1023978 (PMC9907085; doi:10.3389/fcimb.2022.1023978)
Supplement: Supplementary Table 1 — Conventional microbiological methods used in the study. [file Table_1.docx]

**Table S1.** Conventional microbiological methods used in the study.

| Pathogen | Technique | Sample | **Microbiological methods/tests** |
| --- | --- | --- | --- |
| **Bacterial** | Bacterial culture | BALF/blood /sputum | Bacterial culture on appropriate media with bacterial identification by MALDI-TOF mass spectrometry |
|  | Smear | BALF/sputum | Acid-fast staining for *Mycobacteria* |
|  | Antigen detection | Urine | *Legionella* and *pneumococcal* antigen |
|  | Serological diagnosis | Blood | T-SPOT for *Mycobacterium tuberculosis* |
| **Fungal** | Fungal culture | BALF/blood /sputum | Fungal culture on appropriate media with fungal identification by MALDI-TOF mass spectrometry |
|  | Smear | BALF | India ink staining for *Cryptococcus*; gomori methenamine staining for *Pneumocystis* |
|  | Antigen detection | Blood | Cryptococcal antigen, galactomannan and 1,3-D glucan antigen |
|  |  | BALF | Cryptococcal antigen and galactomannan antigen |
| **Viral** | Antigen detection | NP swab | Influenza A/B antigen |
|  | PCR | BALF | Influenza A/B, Epstein-Barr virus and Cytomegalovirus |
| **Atypical pathogens** | Serological diagnosis | Blood | *Mycoplasma pneumoniae* and *Chlamydia pneumoniae* |

BALF, bronchoalveolar lavage fluid; MALDI-TOF, matrix-assisted laser desorption/ionization-time of flight; T-SPOT, T cell spot test of tuberculosis infection; NP, nasopharyngeal; PCR, polymerase chain reaction
